# Experiences of distortions to the passage of time during the Argentinian Covid-19 pandemic

**DOI:** 10.1371/journal.pone.0266261

**Published:** 2022-03-31

**Authors:** María Elena Brenlla, Guadalupe Germano, Mariana S. Seivane, Rocío Fernández da Lama, Ruth Ogden

**Affiliations:** 1 Centro de Investigaciones en Psicología y Psicopedagogía (CIPP), Universidad Católica Argentina, Buenos Aires, Argentina; 2 School of Psychology, Liverpool John Moores University, Liverpool, United Kingdom; University of Connecticut, UNITED STATES

## Abstract

The Coronavirus-19 global pandemic has forced many governments around the world to enforce “lockdowns” to curtail the spread of the virus. Studies conducted in the UK, France, Italy and Brazil have demonstrated that one consequence of these lockdowns is significant distortion to the speed of the passage of time. The current study sought to establish how the passage of time was experienced during the Argentinian lockdown. An online questionnaire was used to measure passage of time judgments for the day and the week, physical activity, satisfaction with social interaction, the extent to which daily routines had changed due to covid and demographic data. The results show that distortions to the passage of time were widely experienced during the lockdown in Argentina. There was a tendency for participants to report time passing more quickly than normal. A faster passage of time was associated with being a woman, of younger age and more physically active. A slower passage of time was therefore associated with being a man, of older age and less physically active. The results indicate that whilst distortions to the passage of time during the covid-19 crisis appear to be a global phenomenon, cross-cultural differences are apparent in the factors which influence temporal experience.

## Introduction

In the wait for sufficient levels of vaccination against the novel coronavirus-19, governments across the globe have attempted to control the spread of the virus by imposing restrictions on the movements and activities of residents. These restrictions are commonly referred to as lockdowns or stay at home orders. The primary aim of a lockdown is to limit physical contact between individuals from different households to reduce opportunities for the virus to spread between households. As a result, they often include limits on who can go to work, who can go to school, which commercial (i.e. none essential shops) and social (i.e. restaurants) enterprises can remain open and how many people can meet indoors and outdoors in a group. As a result of covid-19 lockdowns, residents have experienced significant disruption to all elements of daily-life, often for significant periods of time.

Such significant changes to normal daily life have influenced the way in which people perceive the world around them. In particular, there is growing evidence that the disruption to daily life has distorted the subjective speed of the passage of time for many [1–9]. Although objectively time passes at a constant linear rate, subjectively time can sometimes feel like it is passing more quickly or slowly than normal, resulting in the sensation of time speeding up or dragging by [10, 11]. How we experience the passage of time can be assessed using passage of time judgments (POTJ). POTJs are typically likert or numerical rating scales which ask participants to indicate the speed at which they feel like time is passing in comparison to normal. Passage of time judgments are therefore subjective experiential ratings of the speed of time rather than objective assessments of time itself.

Studies indicate that distortions to the subjective passage of time have been widely experienced during the global pandemic [1–9]. In the UK, for example, studies conducted during the first and second UK lockdowns revealed that 80% of people reported experiencing some sort of distortion to the speed at which time passed during the lockdowns in comparison with “normal” i.e. prior to lockdown [1, 2]. Significant distortions to the subjective speed of time have also been reported in France [3, 4], Italy [5, 6] and Brazil [7]. There is also evidence of increased time distortion for patient groups such as dementia with Lewy Bodies [6]. The sensation of covid-19 induced time distortion therefore appears to be a widespread phenomenon.

Although distortions to the passage of time during covid-19 appear common, there are notable differences in the nature of the distortions observed in the different countries in which they have been studied. In the UK, for example, when considering the passage of time during the day and week during the pandemic, of the 80% of participants who reported distortion to the passage of time, approximately half reported that time felt like it was passing more quickly during the lockdown than before the lockdown (i.e. normal). The remaining half reported that time felt like it was passing more slowly than normal during lockdown [1, 2]. Despite this, when participants were asked about a longer epoch (whether it felt like longer or shorter than 8 months since the first UK lockdown) the majority of participants reported that it felt longer, suggesting a slowing of time when considering the full duration of the pandemic [2]. In France however, for the majority of people, the average subjective speed of time was perceived to have slowed from prior to lockdown to the present moment, the day and the week during lockdown [3, 4]. Similarly, in Italy, typically developing participants overwhelmingly experienced a slowing of the passage of time during their initial period of lockdown [5]. A slowing of time during lockdown in comparison with normal was also observed in a study of time experience in Italian patients with dementia with Lewy bodies [6]. Finally, a longitudinal study conducted in Brazil [7], revealed that at the start of the pandemic 65% of participants reported experiencing an increase in feeling of time expansion in comparison to prior to the pandemic. 75% of participants also reported a decrease in the feeling of time pressure. However, as the period of social isolation in Brazil became more prolonged, the sensation of temporal expansion declined, however the sensation of time pressure remained stable.

Direct comparison between the data collected in Brazil [7] and Europe [1–6] is complicated by the different methods used to assess time experience. The majority of European studies have used likert or numerical rating scales to measure experiences of the relative speed of time during the pandemic in comparison to normal. Although Cellini et al. [5], focused on exploring temporal expansion during boredom, rather than the more general passage of time. The data collected in the Brazilian study however focused on measures of temporal awareness and therefore, although time expansion may be associated with a slowing of the passage of time and time pressure a speeding up of the passage of time the measures are not directly comparable. Regardless of measurement technique, it is however clear that whilst experiences of distortion to the passage of time appear to be a global phenomenon, the directionality of the distortion varies from country to country.

In addition to cross-cultural differences in the nature of temporal experience during the pandemic there also appear to be some territorial differences in the factors which predict the experience of distortions to the passage of time. In the UK’s first lockdown, for example, the subjective speed at which time passed during the day was predicted by age, satisfaction with social interaction, task-load and stress. Lockdown days passed more quickly with increasing task-load, increasing satisfaction with social interactions, reduced stress and reduced age [1]. The days passing more slowly was therefore associated with older age, decreased social satisfaction, decreased task-load and increased stress. Similar findings were also observed for the speed at which the week was perceived to pass, with a faster week being associated with decreasing age and increasing social satisfaction [1]. In the UK’s second lockdown, distortions to the passage of time were predicted by satisfaction with social interaction, depression and shielding (being advised to avoid all contact with others). A slower second lockdown was therefore associated with greater dissatisfaction with social interaction, greater depression and having to shield [2].

In France, distortions to the subjective speed of time were mediated by boredom and sadness, with increases in boredom and sadness being associated with greater slowing of the passage of time [3, 4]. Stress at home, financially or in work, and age were not however predictive of the slowing of time [3, 4]. As in France, in Italy a slowing of the passage of time was associated with greater boredom [5]. Finally, in Brazil [7], experiences of time pressure were predicted by stress and busyness, with increased stress and increased working hours being associated with greater time pressure. The experience of time expansion was predicted by affective factors, with greater negative affect, reduced positive affect and greater social isolation being associated with time expansion.

Although there are differences in the individual predictors identified in different countries, there are some parallels between some of the factors studied. For example, there are similarities between Ogden’s [1, 2] measure of task-load and the measures of boredom in Droit-Volet et al. [3], and Martinelli et al. [4], and measures of working hours in Cravo et al. [7]. This is because reduced task-load is often associated with an increase in boredom [12, 13]. Similarly, there is likely to be overlap between Ogden’s [1, 2] measure of social satisfaction, Cravo et al.’s [7], measure of social isolation and Droit-Volet et al. (2020) [3], and Marintelli et al. s’ (2021) [4] measure of sadness. Social isolation is associated with increases in negative affect and depression [14–17], indeed Ogden’s (2020) [1] measure of satisfaction with social interaction correlated positively with their measure of depression. Interestingly however, Ogden’s measure of depression itself was not predictive of temporal experience during the first lockdown suggesting that social interaction quality was uniquely predictive of time experience beyond its associated impact on mood.

It is unclear why territorial differences in the subjective speed of the passage of time during lockdown occurred. The use of different measures of subjective time experience in each country coupled with differences in the other variables measured to predict time experience complicate cross-cultural comparison. Nonetheless, one possibility is that they reflect cross-cultural differences between the countries of study. For example, the UK has a greater proportion of elderly people living alone than France and Brazil [18]. This may have contributed to greater levels of isolation in this group, resulting in age being predictive of temporal experience in the UK but not directly predictive in France and not associated with time experience in Brazil.

Another possibility is that the differences in temporal experience reflect differences in the specific lockdown restrictions imposed in each country. Table 1 provides comparison of the primary restrictions imposed during the times of data collection for the studies reviewed above. In addition, cumulative infections and deaths per 100,000 of the population at the start and end of the periods of data collection are reported. Examination of Table 1 confirms cross-country differences in social restrictions. For example, in the UK, residents could leave their homes without a permission slip, in France and Italy however, a signed and dated permission slip was required to leave the house or to travel. In the UK, exercise was permitted anywhere for 1 hour per day, however in France exercise had to be within 1km of your home and could not include cycling (or jogging in Paris before 7pm). The consequences of breaking the rules also differed between the UK, Italy and France in terms of financial penalties. It is therefore possible that the relatively stricter lockdown rules in France and Italy than in the UK may have contributed to the predominant slowing of time observed in these countries.

**Table 1:** Lockdown restrictions, cumulative infections per 100,000 and deaths per 100,000 in the UK, France, Italy, Brazil and Argentina during the times of data collection.

Country	Study	Dates	Lockdown conditions	Cumulative infections per 100,000 [19]	Cumulative deaths per 100,000 [19]
UK	Ogden 2020 [1]	7^th^– 30^th^ April 2020	First full lockdown: Schools and non-essential shops closed. Complete prohibition of indoor and outdoor mixing between households. Movement outside of the home only permitted for (1) shopping for basic necessities, (2) one form of exercise a day, alone or with members of their household, (3) any medical need, (4) travelling for work purposes, but only where they cannot work from home. No declarations or passes required to leave home.	105.31–253.81	11.08–39.17
	Ogden 2021 [2]	11^th^ -30^th^ November 2020	Second full lockdown; Schools remained open Work from home recommended wherever possible. Non-essential shops closed, People were required to stay at home wherever possible. Household mixing was prohibited indoors in all circumstances and outdoors in most circumstances.	1843.90–2410.83	71.75–85.76
France	Droit-Volet et al. [3]	31^st^ March– 12^th^ April 2020	First full lockdown: Closure of schools, universities and non-essential public places (shops, cinemas). Written declaration required to leave the house for essential purposed i.e. grocery shopping and seeking medical attention. This could only occur for up to 1 hour per day and travel was only permitted within 1km of residence. Financial penalties for breaching rules.	87.76–165.91	5.23–21.35
	Martinelli et al. [4]	17^th^ March– 11^th^ May 2020		11.44–264.96	0.22–39.52
Italy	Cellini et al. [5]	24-28^th^ March 2020	First full lockdown: Closure of schools, universities and non-essential public places (shops, cinemas etc.). Working from home mandated, travel outside of local area prohibited. Fines and prison sentences imposed for non-compliance.	114.59–153.18	11.30–16.60
	Torboli et al. [6]	15^th^ April-10^th^ July 2020	Control data collected during full lockdown with the rules stated for Cellini et al. [5]. DBL group data collected when no lockdown was imposed.	273.58–401.93	35.86–57.88
Brazil	Cravo et al. [7]	6^th^ May– 21st August 2020	Social isolation protocols were not centralized by the Federal government and thus varied across regions in their implementation dates and restrictiveness.	59.17–1654.57	4.01–53.06
Argentina		7^th^– 17^th^ September 2020	Lockdown phase 3, 5.5 months after the first lockdown. Geographical segmentation according to epidemiological criteria, taking into account the number of infections per day per province. Essential workers could move freely around the country. Commercial and recreational activities were allowed. Population mobility reached up to 50% and public transport coverage was expanded.	1939.31–2085.04	22.21–27.32

To fully understand the impact of Covid-19 and the associated social restrictions imposed during the pandemic on the experience of time is it important to study temporal experience in a broader range of countries. The current study therefore sought to establish how the Argentinian lockdown affected the experience of the passage of time for Argentinian residents.

The first case of covid-19 in Argentina was reported on 3^rd^ March 2020 and the first death on the 7^th^ March 2020. In response to rising cases and deaths a national lockdown was imposed on the 19^th^ March 2020. Restrictions initially included the prevention of cultural, recreational, sporting, religious, or any other type of event that involved people attending, the closure of non-essential shops and businesses and the restriction of movements for all but essential workers. The severity of the restrictions imposed in Argentina had a significant impact upon the population and the economy. In 2020, GDP declined by 9.9% in 2020 and inflation rose to 36% [20]. Consequently, there was also a 6.5% rise in poverty from 2019 to 2020 [20]. Following a fall in deaths and cases, restrictions were progressively eased, first outside of the Greater Buenos Aires area on the 10th of May 2020 and then within Greater Buenos Aires on the 17th of July 2020 [21]. This easing enabled some businesses to reopen (e.g. beauty salons, gyms, bars and restaurants) and a more normal form of life to resume, however social distancing and limits on movement were still present.

The significant changes to daily life resulting from the Argentinian government’s response to covid-19 means that it is plausible that Argentinians may have experienced widespread distortion to the passage of time during the global pandemic, as has been observed in the UK, France, Italy and Brazil. However, differences in the specific lockdown restrictions imposed in Argentina and the UK, France, Italy and Brazil (see Table 1 for summary), and in particular the relatively greater levels of freedom available to Argentinians at the time of study, may have protected the population from experiencing distortions to the passage of time. Furthermore, cross-cultural differences between Argentina, the UK, France, Italy and Brazil may also influence the manifestation of any distortions to time observed in Argentina. For example, Argentina has a higher birth-rate than the UK, Italy, France and Brazil [22], and a lower life expectancy [23] and a lower proportion of elderly people living alone than the UK, France and Italy [18]. As a result, average household size is greater in Argentina than in the UK, France and Italy [24] which may lead to less social isolation, particularly in the elderly for Argentinians than their European counterparts. Greater opportunity for social interaction due to less restrictive lockdown practices and demographic factors may therefore mitigate the effect of the pandemic on the passage of time in Argentina.

To establish how subjective the subjective speed of the passage of time was experienced during the Argentinian lockdown, the current study used a translated version of the online questionnaire developed in Ogden (2020) [1]. This questionnaire asked participants to rate how quickly they felt the day and the week were passing in comparison to prior to the start of lockdown in Argentina. The questionnaire also explored demographic factors such as perceived risk from covid, gender, cohabitation status and employment status, levels of satisfaction with social, levels of physical activity, interaction and the extent to which daily routines had changed during covid-19. The questionnaire was delivered 5.5 months after the first lockdown was imposed during which the country was divided into geographical segmentations according to epidemiological criteria, taking into account the number of infections per day per province. Essential workers were however able to move around the country freely. Commercial and recreational activities were reopened, including most shops, gyms, beauty salons, bars and restaurants. As a result, population mobility returned to half of pre-pandemic levels and public transport coverage was expanded.

Based on the findings of existing research conducted in the UK [1, 2], France [3, 4] Italy [5, 6] and Brazil [7], it was expected that participants would report experiencing the subjective passage of time during the pandemic as different to before the pandemic. Widespread distortion to the passage of time was expected because, at the time of study, covid-19 restrictions were still having a significant effect on daily life in Argentina. However, because lockdown restrictions were less strict in Argentina at the time of study than in other countries, and because of existing demographic and economic difference between Argentina and other counties in which time experience during covid has been studied, it was not possible to develop specific hypotheses about the predominant direction in which any distortions to the passage of time would manifest.

It was also expected that age would be related to the subjective speed of the passage of time, with older Argentinians experiencing a slower passage of time during the pandemic than younger Argentinians. This hypothesis was based on previous pandemic research showing a negative relationship between age and the passage of time [1–3] and existing research conducted prior to covid-19 demonstrating that the passage of time slows in the very elderly [25]. In addition to these hypotheses, exploratory analyses were also conducted to explore the effects of demographics, social satisfaction, physical acitvity and change of daily routine on experiences of the passage of time.

## Method

### Participants

1229 participants were recruited through snowball sampling via email and social media advertising. 51 were excluded from the study because they were younger than 18 years old, or because they failed to answer one or more questions. This left a final sample of 1185 participants. Table 2 shows demographic information. The study was approved by Liverpool John Moores University Research Ethics Committee (ref 20/NSP/01) and all participants gave informed written consent. The study was conducted in accordance with the principles expressed in the Declaration of Helsinki.

**Table 2:** Descriptive statistics for demographic information.

Age	MIN	18
	MAX	78
	*M* (SD)	34.00 (14.86)
Gender	Female	63.60% (*n* = 748)
	Male	36.10% (*n* = 428)
	Other	0.3% (*n* = 4)
Currently working	Yes	64.10% (*n* = 760)
	No	35.90% (*n* = 425)
Cohabitation	Living alone	11.10% (*n* = 131)
	Cohabiting	88.90% (*n* = 1054)

### Measures

Participants completed an online questionnaire distributed through google forms. The questionnaire was a modified and translated version of that used in Ogden (2020) [1]. The questionnaire recorded demographic information, perceived risk from covid, passage of time judgements, satisfaction with social interactions, average level of physical activity and the extent to which daily routine had changed as a result of covid-19. The questionnaire was released to participants on the 7th of September 2020, 5 months and a half after the commencement of lockdown, and closed 17th of September 2020. At the time that the questionnaire was released most of the population had been in isolation for almost 6 months. Participants took approximately 10 minutes to complete the whole questionnaire.

### Demographic questions

Participants stated their age, gender, how many people they lived with, employment status and whether they were in a high-risk category for Covid-19.

### Passage of time judgements

The following questions were posed about the daily and weekly passage of time.

1. *“Thinking about today*, *how quickly time has felt like it is passing in comparison with normal (i*.*e*. *before lockdown)*?*”*
2. *Thinking about this week*, *how quickly has time felt like it was passing in comparison to normal (i*.*e*. *before lockdown)*?

Participants responded using the following 7-point Likert scale: 1. Extremely slow, 2. somewhat slower, 3. a little slower, 4. as normal, 5. a little faster, 6. somewhat faster, 7 extremely fast. A higher score therefore indicated a faster passage of time.

### Social satisfaction, physical activity and change in daily routine

To measure social satisfaction participants were asked to rate how “Since the Covid-19 lockdown, how satisfied are you with your daily level of social interaction?” using a 5 point Likert scale in which a high score indicated greater dissatisfaction. To measure physical activity, participants rated “Since the Covid-19 lockdown, how would you describe your level of physical activity? Using a 5 point Likert scale in which a high score indicated greater inactivity. Finally, participants also used a 5 point Likert scale to rate to what extent they agreed that: "My daily routine has changed a lot as a result of the Covid-19 lockdown? Here, a high score indicated greater disagreement, so lower scores indicate participants feel their routine changed.

### Data analysis

Because the main dependent variables were ordinal scales, nonparametric analyses were conducted. As in Ogden (2020) [1], age was classified into three groups: young adults (25 years and under), middle aged adults (26–59 years) and older adults (aged 60 years and over). Kruskal-Wallis and Mann Whitney U tests were used to establish the effect of gender, age group, cohabitation status, employment status and perceived personal risk on POTJ-day and POTJ-week. To assess the relationship between the passage of time, age and measures of satisfaction with social interaction, physical activity and change to daily routine, Spearman’s correlations were conducted. For these tests, correction for multiple comparisons was made using the Bonferroni correction for each analysis. Finally, to establish the predictors of distortions to the passage of time, ordinal regression analysis was conducted.

## Results

**Fig 1:**
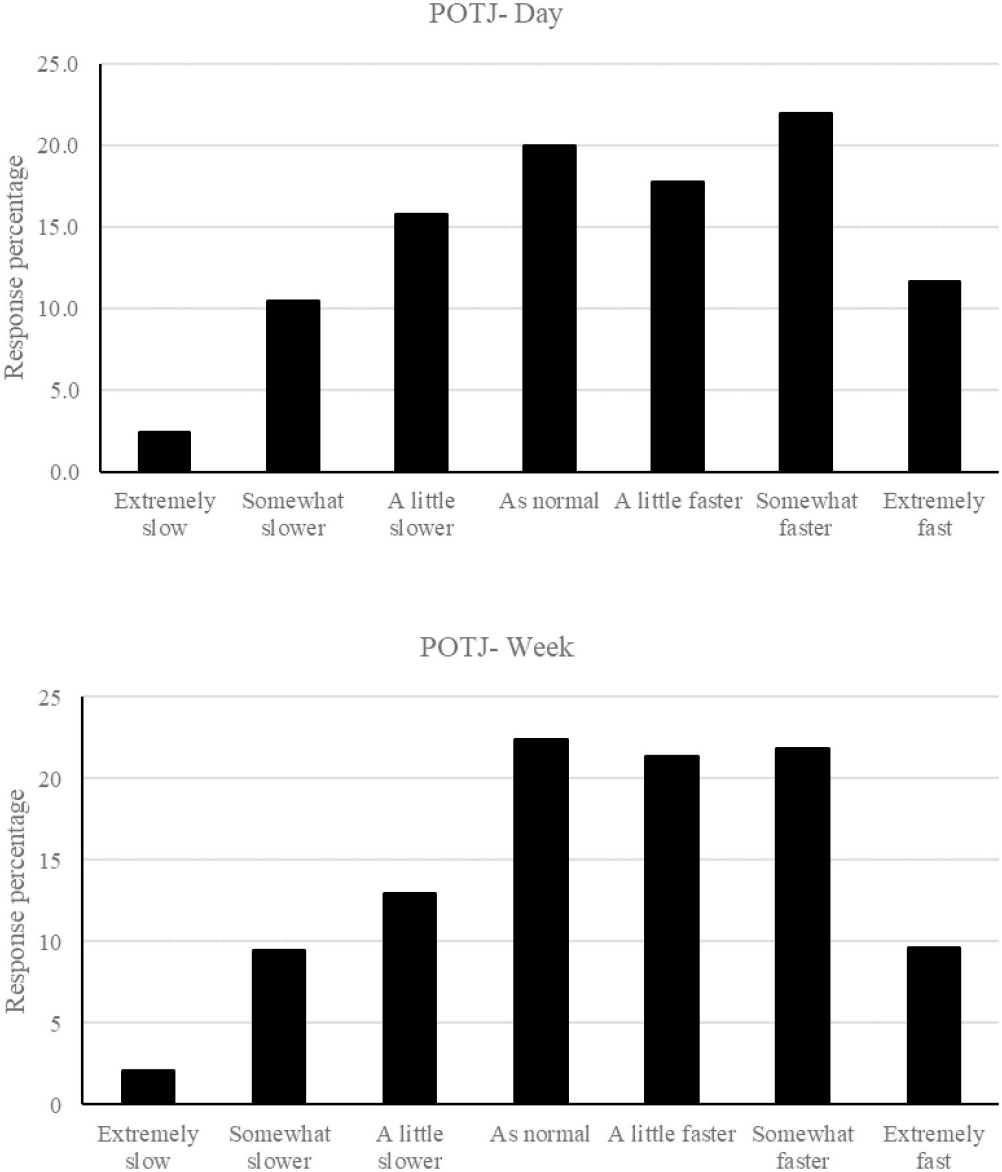
The frequency of responses for each Likert point for the day passage of time judgement (upper panel) and week passage of time judgment (lower panel).

Fig 1 shows the distribution of responses for the day (upper panel) and week (lower panel) passage of time judgments. Examination of Fig 1 suggests that reports of distortion to the passage of time were highly prevalent in Argentina during the period of lockdown. For POTJ-day, 80% of the participants reported some distortion to the passage of time during lockdown in comparison with normal. 29% of participants reported experiencing the sensation of a slowing of time during the day relative to prior to lockdown, whereas 51% experienced a speeding up of the passage of time. For POTJ-week, only 22% of participants reported no distortion in the passage of time for the week during lockdown in comparison to prior to lockdown. 25% of the participants reported that time was passing more slowly than normal across the week, however 53% reported that time felt like it was passing more quickly than normal across the week. Spearman’s Rho correlation confirmed a significant positive relationship between POTJ-day and POTJ-week *r*(1185) = .74, *p* < .001.

To explore the effects of demographic factors on experiences of the passage of time, the effects of age group, gender, personal risk, cohabitation stats and employment status were explored. Table 3 shows mean passage of time judgments expressed as a function of these factors.

**Table 3:** The mean POTJ for each displayed as a function of age, gender, employment, personal risk and cohabitation status.

		Mean POTJ-day (SD)	Mean POTJ–week (SD)
Age	Young < 26 (N = 543)	4.70 (1.64)	4.68 (1.52)
	Middle aged (N = 576)	4.43 (1.61)	4.51 (1.54)
	Older ≥60 (N = 66)	3.95 (1.39)	3.95 (1.42)
Gender	Female	4.64 (1.64)	4.75 (1.55)
	Male	4.32 (1.57)	4.33 (1.49)
Personal Risk	Yes	4.56 (1.64)	4.56 (1.55)
	No	4.61 (1.66)	4.63 (1.69)
	Unsure	4.38 (1.54)	4.47 (1.40)
Cohabitation status	Cohabiting	4.52 (1.77)	4.51 (1.59)
	Living Alone	4.53 (1.60)	4.56 (1.53)
Employment	Employed	4.56 (1.66)	4.57 (1.58)
	Unemployed	4.51 (1.60)	4.55 (1.51)

Data is show separately for the POTJ-day and POTJ-week.

### Age

There was a significant effect for age group on POTJ-day (*X*_(2)_ = 17.62, *p* < .001). Post-hoc Mann-Whitney U tests (Bonferroni corrected) confirmed a significantly faster perceived passage of time in the young group than the middle-aged group (*p* = .004) and elderly group (*p* < .001). There was no significant difference in POTJ-day for middle-aged group and the elderly group (*p* = .015). There was also a significant effect of age group on POTJ-week (*X*_(2)_ = 17.08, *p* < .001). Post-hoc Mann-Whitney U tests (Bonferroni corrected) confirmed a significantly faster perceived passage of time in the young than the elderly group (*p* < .001) and the middle-aged group than the elderly group (*p* = .004). There was no significant difference in POTJ-week for the young and middle-aged groups (*p* = .03).

### Gender

Due to the small number of responses from participants who did not select male or female for their gender (n = 4) these responses were excluded from analysis. There was a significant effect of gender on POTJ-day (*U* = 141452.50, *p* = .001) and POTJ-week (*U* = 138320.50, *p* < .001) with the passage of time being experienced as subjectively quicker for females than males.

### Personal risk

There was no effect of personal risk from COVID-19 on POTJ-day (χ^2^_(2)_ = 4.39, *p* = .11) or POTJ-week (χ^2^_(2)_ = 3.27, *p* = .20).

### Cohabitation status

There was no effect of cohabitation status on POTJ-day (U = 68530.00, *p* = .90) or POTJ-week (U = 67776.50, *p* = .74).

### Employment status

There was no effect of employment status on POTJ-day (U = 157632.00, *p* = .49) or POTJ-week (U = 157791.00, *p* = .55).

### The relationship between the passage of time and measures of social satisfaction, physical activity, age, gender, risk and cohabitation

Table 4 shows correlation coefficients for the relationships between POTJs and age, measures of social satisfaction, physical activity and change to daily life. There were significant negative relationships between POTJ-day, POTJ-week and age suggesting that time was experienced as passing more slowly with increasing age. There were no significant relationships between the extent to which daily routine had changed, levels of social satisfaction, physical activity and POTJ-day and POTJ-week. There were however significant positive relationships between age and social satisfaction. Social satisfaction therefore increased with increasing age. Social satisfaction was negatively related to change to daily routine and positively related to physical activity.

**Table 4:** Correlation coefficients between POTJs, age, measures of social satisfaction, change to daily life and level physical activity.

	POTJ-day	POTJ- week	Age	Social satisfaction	Change to life
**Age**	-.16**	-.15**			
**Social satisfaction**	-.04	-.04	.08**		
**Change to daily life**	.03	.02	.02	-.23**	
**Physical activity**	-.05	-.05	.06	.16**	.02

**p < .001.

Ordinal regression with proportional odds was conducted to establish the effect of demographic factors, social satisfaction, physical activity and change of life on POTJ-day and POTJ-week. Table 5 shows the odds ratios for each variable with 95% confidence intervals.

**Table 5:** Wald, odds ratios and 95% confidence intervals from the ordinal regressions for POTJ-day and POTJ-week.

		POTJ-Day			POTJ-Week		
		*Wald*	*Odds Ratio*	*95% CI*	*Wald*	*Odds Ratio*	*95% CI*
*Age*		28.03	-.02**	-.03–-.01	21.94	-.0**	-.03–-.01
*Gender*	Other	.2.35	1.38	-.39–3.16	2.97	1.56	-.22–3.34
	Female	10.34	.35**	.14 -.56	14.40 7	.42**	.20 -.63
	Male (reference)						
*Cohabitation status*	Alone	.02	.25	.-.30-.35	.12	-.06	--.39–.27
	Cohabiting (reference)						
*Perceived greater risk*	Yes	3.14	.22	-.03 -.47	1.15	.14	-.11–.38
	No	1.48	.17	-.11–.45	1.15	.15	-.13–.43
	Unsure (reference)						
*Employment status*	No	2.67	-.19	-.42–.04	2.88	-.20	-43–.03
	Yes (reference)						
*Socialisation satisfaction*		.66	-.04	-.13–.06	.47	-.03	-.13–.06
*Physical activity*		3.17	-.08	-.18–.01	4.10	-.10*	-.19–-.01
*Change of routine*		1.95	.09	-.42–.04	1.84	-.20	-.43–.03

**p < .001;

* *p* < .05.

For the POTJ-day, the model was a statistically significant, χ2(10) = 52.76, *p* < .001 fit for the data, with pseudo R squared values of .01–.05. Age was predictive of POTJ-day, with increasing age being associated with a slowing of the passage of time. Gender was also predictive of POTJ-day with being female being associated with a faster passage of time. Cohabitation status, perceived personal risk, employment status, satisfaction with social interaction, physical activity and change to daily routine were not predictive of POTJ-day.

For the POTJ-week, the model was a statistically significant, χ2(10) = 49.76, *p* < .001 fit for the data with pseudo R squared values of .01–.05. Age was predictive of POTJ-week, with increasing age being associated with a slowing of time across the week. Gender was also predictive of POTJ-week with female gender being associated with time feeling like it was passing more quickly than normal. Physical activity was also predictive of POTJ-week with greater physical activity being associated with reports of a faster passage of time. As with POTJ-day, cohabitation status, perceived personal risk, employment status, satisfaction with social interaction and change to daily routine were not predictive of POTJ-week.

## Discussion

This study sought to measure experiences of the passage of time during the Argentinian lockdown. The results showed that experiences of distortions to the passage of time during the lockdown were highly prevalent. Fewer than 25% of people reported that time passed as normal during the lockdown for the POTJ-day and POTJ-week. This confirms the hypothesis that time would be experienced as subjectively different during the pandemic relative to before. It also findings of studies conducted in the UK [1, 2], France [3, 4], Italy [5, 6] and Brazil [7] which also observed widespread reports of distortions to the passage of time during their country’s lockdowns. Feeling as though time is distorted during a covid-19 lockdown therefore appears to be a global phenomenon experienced across cultures.

Unlike in France [3, 4] and Italy [5, 6], in which the passage of time was perceived to be slower during lockdown than before, and unlike in the UK [1, 2] in which time was equally likely to feel like it was passing more quickly or more slowly than normal, in Argentina, the experience of time passing more quickly than before the pandemic was more commonly reported than time passing more slowly. For POTJ-day, 51% of participants reported experiencing time as passing more quickly than normal compared with 29% for more slowly than normal. For POTJ-week, 53% of participants reported the week as passing more quickly during covid than before, in comparison with 25% for more slowly. Argentina therefore appears to be unique in the sense that more people experienced an acceleration of the passage of time during lockdown than a slowing of the passage of time.

Analysis of the factors which predicted the experience of distortions to the passage of time during the day revealed age and gender to be predictive factors. The day passing more quickly than normal was associated with decreasing age and being female. Conversely, the day passing more slowly than normal was associated with male gender and increasing age. For POTJ-week, age, gender and physical activity were predictive factors. The experience of the week passing more quickly than normal was associated with decreasing age, female gender and increased physical activity. Conversely the experience of the week passing more slowly than normal was associated with increasing age, male gender and reducing physical activity. Satisfaction with social interaction, the extent to which life had changed due to covid-19, personal perceived risk from covid-19 and cohabitation status were not predictive of POTJ-day or POTJ-week.

The association between age and distortions to the passage of time in Argentina confirms the hypothesis that time would be experienced as passing more slowly for older than younger individuals. It also replicates findings from Ogden’s (2020) [1] study of the UK’s first lockdown in which time was also found to pass more slowly for people over the age of 65 years. These findings are also partially supported by observations of a negative relationship between age and the speed of the passage of time in France [3]. Although a slower passage of time for the elderly than the young was not observed in Brazil, the use of temporal awareness scale in Brazil as opposed to POTJs in UK, France and Argentina may explain this difference [7]. Evidence of a slower passage of time for older residents than younger residents in Argentina, the UK and France suggests that the disruptive effects of lockdown may have had a particularly significant effect on time experience for the elderly. This may reflect greater levels of loneliness and isolation [26] and increases in anxiety and reductions in mental capacity [27] in elderly than younger people during lockdown. These findings, coupled with those showing that the passage of time also slows down for elderly people when they enter residential care [28] confirms the importance of understanding time experience in later life. Future research should therefore seek to identify the precise circumstances in which time distorts for older individuals.

The exploratory analysis of the influence of demographic factors on experiences of the passage of time revealed an association between female gender and the experience of a faster passage of time and male gender and a slower passage of time. These associations appear to be unique to Argentina as gender was not observed to be a predictive factor in temporal experience in the UK, Italy, France and Brazil. One explanation for this may be that studies examining the effect of the effect of lockdown restrictions on the distribution of paid and unpaid (i.e. domestic duties) labor for males and females suggest that in Argentina, females have experienced a disproportionate increase in workload, particularly unpaid workload, in comparison with males [29]. Increased busyness and workload have previously been associated with a faster passage of time, in part because attention is dedicated to task completion rather than monitoring the passage of time [10, 30–34]. It is therefore possible that the disproportionate increase in workload experienced by Argentinian females contributed towards their experience of a faster passage of time. However, there is also evidence that the workload of females has been disproportionally affected by lockdown in comparison with that of men from studies conducted in the UK [35, 36], France [37], Italy [38] and Brazil [39] however there was no evidence of gender influencing the passage of time in these countries. It is therefore unlikely that changes in workload for Argentinian females is solely responsible for the faster experience of time for Argentinian females than males.

The association between female gender and a faster passage of time in Argentina and male gender and a slower passage of time may however be explained by the effect of covid on role assignment in Argentina. In ordinary times, many males and females in Argentina have to deal with long commutes and lengthy workdays. In addition, females are often disproportionately impeded by the *unpaid work* phenomenon and work-related setbacks when it comes to asking for leave and work permissions. It is therefore possible that, although the Covid pandemic forced people to readapt to an unexpected situation, it also meant in many cases an easing of many practical and family-related issues for women (such as not having to use public transportation and a more equal divide in household chores between the family members). As a consequence, however, males may have had to take up tasks that were previously and routinely “delegated” to their partners. Data from the Organization for Economic Co-operation and Development [OECD] data suggests that English males are more accustomed to unpaid care work than Argentinian males [40]. The greater effect of the pandemic on male activities in Argentina than in the UK may therefore have contributed gender influencing experiences of the passage of time in Argentina but not the UK.

A further possible explanation for the effect of gender on time experience in Argentina is that the lockdown had different effects on mental health and wellbeing for males and females. In particular, Argentinian males appeared to experience greater falls in positive affect than females [41]. Indeed, the technical report of the Argentine Social Debt Observatory (which assesses different human development aspects year-to-year, including psychological distress), shows that—during lockdown—the percentage of females reporting anxious and depressive symptoms decreased in comparison to 2019 [42]. Affect is known to alter the passage of time, with greater levels of positive affect being associated with a faster passage of time and increased negative affect being associated with a slowing of the passage of time [10, 30, 43]. Time may therefore have slowed to a greater extent for Argentinian males than females because they experienced a greater fall in positive affect. However, if gender differences in mental wellbeing were a significant contributing factor in gender differences in time experience, it is unclear why comparable effects were not observed in Brazil and the UK where females have experienced greater declines in mental wellbeing than males [39, 44]. Further research should therefore seek to understand the precise causes of gender differences in experiences of the passage of time.

The absence of an effect of social satisfaction on experiences of distortions to the passage of time during covid-19 in Argentina contrasts markedly with findings from the UK and Brazil where social satisfaction and isolation were key predictors of distortions to time in those countries. One possible explanation for this is that lockdown conditions were more relaxed in Argentina than in the UK and Brazil at the times of study. During the current study, Argentina was experiencing a “chronic” but not strict lockdown, with few restrictions regarding mobility, social meetings with people outside the household and group physical or social activities. At the time of studies in the UK, France and Italy, strict lockdowns were in place which prevented almost all forms of face-to-face socialization with people outside of your household. This included mandated working from home where possible, restrictions on exercise, and prohibition of leaving home unless it was to seek essential groceries and medical care. It is therefore possible that increased opportunities for “normal” social interaction in Argentina, in comparison with the UK and Brazil, explains the differing roles of social satisfaction in time experience in these countries. When social interaction is abundant and relatively unrestricted, access to satisfying social interaction therefore appears to have a minimal impact on temporal experiences. However, when social interaction is heavily prohibited, the extent to which it is satisfying is predictive of how we experience the passage of time.

The differences between lockdown restrictions between countries could also explain why physical activity was predictive of POTJ-week in Argentina but not in other countries. The data gathered in Argentina shows that the perception of a faster passage of the week was associated with greater levels of physical activity. This relationship is consistent with previous research showing that a goal-oriented activity is related to the acceleration of passage of time [45]. As there were no restrictions on physical activity in Argentina at the time of study but there were significant restrictions on exercise opportunities in the UK, France and Italy, the lack of opportunity for exercise in Europe may have prevented variability in exercise activity from significantly affecting the passage of time.

Although many of the differences in way in which the passage of time was experienced in Argentina and other countries can be explained by differing lockdown conditions, it is also possible that cross-cultural differences in the way in which time is conceptualised [46] may have contributed to the effects observed. Hall’s (1959) [47] classic ideas regarding the concepts of monochronic time (M-Time) and polychronic time (P-Time) could be of interest to understand these findings. According to Nonis, Teng & Ford’s (2005) [48] research, M-Time cultures conceive time in a linear and analytical way, which leads them to privilege sequentiality. On the other hand, P-Time cultures conceive time in a recurring way and put an emphasis on simultaneity. M-Time cultures are typically associated to developed nations [49], where time tends to be conceptualized in terms of money. Therefore, time not dedicated to working in a task or specific objective is considered to be lost and undesirable time. P-Time cultures however, live in a natural flow of time and focus on human interaction, considering time shared with others as a task in itself because it helps build bonds that can be useful in future times [49].

Hall’s suggestions are consistent with Schwarz’s (2008) [50] research, which shows that Latin America has low scores for Orientation, Uncertainty Avoidance, and Future Orientation. Latin American social orders accept life as it comes, acknowledge its variability as an unavoidable truth, and don’t over-stress [50]. In frail Uncertainty Avoidance social orders, individuals are comfortable with vague, difficult or unpredicted circumstances. They are hence lenient toward change. In solid Uncertainty Avoidance social orders (i.e. Western Europe and the US) individuals feel intimidated by incertitude, have a psychological condition for consistency, and therefore struggle with change. This resistance to change is displayed through nervousness, stress, and endeavours to control circumstances. These cross-cultural differences may therefore have contributed towards the effects observed in addition to any local differences in lockdown arrangements. For example, it is possible that greater flexibility in Argentinian conceptualisations of time enabled them to adapt to the changes in daily life resulting from lockdown more effectively than people in the UK and France (M-time). Indeed, in a period in which people had more time to spend with their cohabitants outside of work (i.e. less commuting, more working from home, closure of schools), the focus on a natural flow of time and simultaneity in P-time cultures may have shaped time experience to feel like it passed more quickly for Argentinians. Similarly, the removal of separation, sequentially and order from daily life for many Europeans (i.e. work and home are now the same location, work and school occur simultaneously in the same location not separately) may have contributed to the slowing of time observed in France and Italy and for many in the UK.

### Limitations

The current study required participants to make a comparison between their memory of how time passed before lockdown with how time was passing now. At the time of the current study, most Argentinians had been in some form of lockdown for 5.5 months, and as a result, memories of what a “normal” passage of time was could have suffered decay and distortion during that retention period, altering the comparison between current temporal experience and normality. However, it should be noted that research examining the effect of ageing on time experience has often used substantially longer comparison periods (e.g. how fast does time feel like it is passing now in comparison with 10 years ago) [51–53]. Future research should therefore try to establish how differing comparison periods affect the reporting of distortions to the passage time.

A related issue is the absence of a baseline data collection point prior to the start of the first lockdown. The absence of a direct comparison of experiences of the speed of the passage of time prior to lockdown with during lockdown means that it is not possible to exclude the possibility that participants would report distorted POTJ’s even in non-pandemic times. Indeed, it should be acknowledged that even in times of “normality” (i.e. prior to the pandemic), some studies report that people frequently experience time as passing more quickly or slowly than normal [50, 53, 54]. The effects reported in this study and others using similar methods should therefore be understood to refer to subjective perceptions that the speed of time has changed in comparison to some memorized speed of time at a previous point in time rather than direct evidence of a change in POTJ before and after the pandemic.

The current study only examined a small number a potential predictor variables. As a result, the proportion of variance in the passage of time explained by the recorded measures is small. It is therefore likely that a broader survey, which incorporated a wider range of measures, would highlight further factors which determine temporal experience in Argentina and explained a greater proportion of the variance in time experience. Given that other studies have indicated that sadness, boredom, task-load and stress all influence the passage of time during lockdown [1–6], and that non-lockdown studies also suggest that emotion and arousal can influence the passage of time [10, 30, 43], it is important that future work examines the potential influence of these factors. However, for future cross-cultural studies, emphasis should be placed on understanding how cross-cultural differences in pace of life and the scheduling of events [46] influence the experience of the passage of time.

A further issue is that although this study observed an effect of gender on temporal experience, there was significant imbalance in gender representation within the sample. Specifically, females represented 63% of the sample. It is therefore possible that under-representation of males in the sample may have influenced the gender effects observed. It is also possible that the greater representation of females in the sample reflects a diffuse self-selection bias which in turn influenced the gender effects observed.

## Conclusions

The findings of this study, coupled with those reported in the UK [1, 2], France [3, 4], Italy [5, 6] and Brazil [7], suggest that feeling like the passage of time has distorted is a common aspect of lockdown life. Regardless of how long a lockdown has been in force for (e.g. weeks in the UK, France and Italy or nearly 6 months in Argentina), or how restrictive the lockdown rules are, many people report experiencing the sensation of a change in the speed of the passage of time relative to before the pandemic. For Argentinians, time was more likely to distort by feeling like it was passing more quickly than normal during lockdown, with a faster passage of time during lockdown being associated with younger age, female gender and greater levels of physical activity. Unlike in the UK and Brazil, social satisfaction was not predictive of temporal experience. The differences in the factors which predicted reports of distortion to the passage of time in Argentina and other countries suggests that societal, cultural and economic factors may influence how and why the passage of time distorts in different countries. However, whilst this and other studies, indicate that people *report* experiencing the passage of time as distorted during the pandemic relative to normal, the presence of distortions to the passage of time in other non-pandemic studies [50, 53, 54] indicates that the subjective experience of time “now” can often feel different to normal/the past. The current findings are perhaps therefore unlikely to represent truly distorted temporal experience, and instead highlight a tendency to report distorted experience now relative to the past.

## Supporting information

S1 File (SAV)

## References

[1] Ogden RS. The passage of time during the UK Covid-19 lockdown. PloS ONE. 2020 Jul 6;15(7):e0235871. doi: 10.1371/journal.pone.0235871 . 32628735 PMC7337311

[2] Ogden R. Distortions to the passage of time during England’s second national lockdown: A role for depression. Plos one. 2021 Apr 20;16(4):e0250412. doi: 10.1371/journal.pone.0250412 33878130 PMC8057617

[3] Droit-Volet S, Sandrine GI, Martinelli N, Andant N, Clinchamps M, Parreira L, et al. Time and Covid-19 stress in the lockdown situation: Time Free, Dying of Boredom and Sadness. PLOS ONE, 2020 Aug 15(8): e0236465. doi: 10.1371/journal.pone.0236465 . 32776990 PMC7416923

[4] Martinelli N, Gil S, Belletier C, Chevalère J, Dezecache G, Huguet P, et al. Time and Emotion in the lockdown for the Covid-19 epidemic: The determinants of our experience of time?. Frontiers in Psychology. 2020;11:3738. doi: 10.3389/fpsyg.2020.616169 33488485 PMC7816521

[5] Cellini N, Canale N, Mioni G, Costa S. Changes in sleep pattern, sense of time and digital media use during COVID-19 lockdown in Italy. Journal of Sleep Research. 2020 May 15:e13074. 10.1111/jsr.13074 PMC7235482 32410272

[6] Torboli D, Mioni G, Bussé C, Cagnin A, Vallesi A. Subjective experience of time in dementia with Lewy bodies during COVID-19 lockdown. Current Psychology. 2021 May 8:1–0. doi: 10.1007/s12144-021-01811-7 33994757 PMC8105146

[7] Cravo AM, de Azevedo GB, Bilacchi CM, Barne LC, Bueno FD, de Camargo RY, et al. Time experience in social isolation: a longitudinal study during the first months of COVID-19 pandemic in Brazil. PsyArXiv; 2021. doi: 10.31234/osf.io/6jg4r PMC9007501 35417245

[8] Wittmann M. (2020). Subjective Passage of Time during the Pandemic: Routine, Boredom, and Memory. *KronoScope*, 20(2), 260–271.

[9] Ogden R. Why Covid-19 might be making us lose our sense of time…. The Cognitive Psychology Bulletin. 2021 Jan 20;6(1).

[10] Wearden JH. Passage of time judgements. Consciousness and Cognition. 2015 Dec 15;38:165–71. doi: 10.1016/j.concog.2015.06.005 . 26115594

[11] Wearden J. The psychology of time perception. Springer; 2016 Jun 9.

[12] Cummings ML, Gao F, Thornburg KM. Boredom in the workplace: A new look at an old problem. Human Factors. 2016 Mar;58(2):279–300. doi: 10.1177/0018720815609503 26490443

[13] Seghi F, Barbini B, Franchini L, Colombo C. The challenge of mental health during Covid-19 outbreak: experience from metropolitan area of Milan. European Archives of Psychiatry and Clinical Neuroscience. 2020 Jun 20:1–2. doi: 10.1007/s00406-020-01154-7 32564126 PMC7305475

[14] Groarke JM, Berry E, Graham-Wisener L, McKenna-Plumley PE, McGlinchey E, Armour C. Loneliness in the UK during the COVID-19 pandemic: Cross-sectional results from the COVID-19 Psychological Wellbeing Study. PLOS ONE. 2020 Sep 24;15(9):e0239698. doi: 10.1371/journal.pone.0239698 32970764 PMC7513993

[15] Brooks SK, Webster RK, Smith LE, Woodland L, Wessely S, Greenberg N, et al. The psychological impact of quarantine and how to reduce it: rapid review of the evidence. The Lancet. 2020 Feb 26. 10.1016/S0140-6736(20)30460-8 PMC7158942 32112714

[16] Banerjee D, Rai M. Social isolation in Covid-19: The impact of loneliness. International Journal of Social Psychology. 2020. 66(6) 525–527. doi: 10.1177/0020764020922269 32349580 PMC7405628

[17] Santini ZI, Jose PE, Cornwell EY et al. Social disconnectedness, perceived isolation, and symptoms of depression and anxiety among older Americans (NSHAP): a longitudinal mediation analysis. Lancet Public Health, 5 (1) 2020, pp. e62–e70. doi: 10.1016/S2468-2667(19)30230-0 31910981

[18] United Nations. Population facts: Living arrangements of older persons around the world https://www.un.org/en/development/desa/population/publications/pdf/popfacts/PopFacts_2019-2.pdf) [Accessed 02/02/2021].

[19] https://ourworldindata.org/coronavirus. https://ourworldindata.org/covid-deaths [Accessed 22/02/2021].

[20] World Bank. The World Bank in Argentina. https://www.worldbank.org/en/country/argentina/overview#1 [Accessed 04/08/2021].

[21] World Health Organisation. Argentina Covid-19 Case Study. file:///C:/Users/rsogd/Downloads/Argentina-C19-case-study-20-May.pdf [Accessed 04/08/2021].

[22] World Bank. Fertility Rates. https://data.worldbank.org/indicator/SP.DYN.TFRT.IN [Accessed on 04/08/2021].

[23] World Health Organization. Life expectancy at birth (years). https://www.who.int/data/gho/data/indicators/indicator-details/GHO/life-expectancy-at-birth-(years) [Accessed 04/08/2021].

[24] United Nations. Household Size and Composition Around the World 2017. https://www.un.org/en/development/desa/population/publications/pdf/ageing/household_size_and_composition_around_the_world_2017_data_booklet.pdf [Accessed 04/08/2021].

[25] Droit-Volet S. Time does not fly but slow down in old age. Time & Society. 2019 Feb;28(1):60–82.

[26] Armitage R, Nellums LB. COVID-19 and the consequences of isolating the elderly. The Lancet Public Health. 2020 May 1;5(5):e256. doi: 10.1016/S2468-2667(20)30061-X 32199471 PMC7104160

[27] Webb L. Covid‐19 lockdown: a perfect storm for older people’s mental health. Journal of Psychiatric and Mental Health Nursing. 2020 Jun 28. doi: 10.1111/jpm.12644 32352621 PMC7267362

[28] Droit-Volet S. Time does not fly but slow down in old age. Time & Society. 2019 Feb;28(1):60–82.

[29] Costoya V, Echeverria L, Edo M, Rocha A, Thailinger A. The impact of COVID-19 in the allocation of time within couples. Evidence for Argentina. 10.1007/s10834-021-09770-8 PMC8138841 34035640

[30] Wearden J, O’Donoghue A, Ogden R, Montgomery C. 14 Subjective Duration in the Laboratory and the World Outside. Subjective time: The philosophy, psychology, and neuroscience of temporality. 2014 Apr 4;4:287.

[31] Wearden JH. The perception of time: basic research and some potential links to the study of language. Language Learning. 2008 Dec;58:149–71.

[32] Larson E, von Eye A. Predicting the perceived flow of time from qualities of activity and depth of engagement. Ecological Psychology. 2006 Apr 1;18(2):113–30.

[33] Flaherty MG. The perception of time and situated engrossment. Social Psychology Quarterly. 1991 Mar 1:76–85.

[34] Flaherty MG. Conceptualizing variation in the experience of time. Sociological Inquiry. 1993 Oct;63(4):394–405.

[35] Oreffice, Sonia and Quintana-Domeque, Climent, Gender Inequality in Covid-19 Times: Evidence from UK Prolific Participants. IZA Discussion Paper No. 13463, SSRN: https://ssrn.com/abstract=3648803.

[36] Office for National Statistics. Parenting in lockdown: Coronavirus and the effects on work life balance. https://www.ons.gov.uk/peoplepopulationandcommunity/healthandsocialcare/conditionsanddiseases/articles/parentinginlockdowncoronavirusandtheeffectsonworklifebalance/2020-07-22 22 July 2020. [Accessed 10/01/2021].

[37] Lambert A, Cayouette-Remblière J, Guéraut É, Le Roux G, Bonvalet C, Girard V, et al. How the COVID-19 epidemic changed working conditions in France. Population Societies. 2020(7):1–4.

[38] Inno L., Rotundi A., & Piccialli A. (2020). COVID-19 lockdown effects on gender inequality. *Nature Astronomy*, 4(12), 1114–1114.

[39] Loret de Mola C, Blumenberg C, Martins RC, Martins-Silva T, Carpena MX, Del-Ponte B, et al. Increased depression and anxiety during the COVID-19 pandemic in Brazilian mothers: a longitudinal study. Brazilian Journal of Psychiatry. 2021(AHEAD). doi: 10.1590/1516-4446-2020-1628 33440402 PMC8136383

[40] OECD. Gender equality in the G20 –Additional analysis from the time dimension. https://www.ilo.org/wcmsp5/groups/public/---dgreports/---cabinet/documents/publication/wcms_713376.pdf [Accessed 10/01/2021].

[41] Canet-Juric L, Andrés ML, Del Valle M, López-Morales H, Poó F, Galli JI, et al. A longitudinal study on the emotional impact cause by the COVID-19 pandemic quarantine on general population. Frontiers in Psychology. 2020 Sep 18;11:2431. doi: 10.3389/fpsyg.2020.565688 33071893 PMC7531077

[42] Rodríguez Espinola S. Filgueira P. Paternó Manavella, M. Recursos Psicosociales Bajo Los Efectos del Ailamiento Social Obligatorio. Informe Técnico -Serie Estudios: Impacto Social de las Medidas de Aislamiento Obligatorio por COVID-19 en el AMBA Observatorio de la Deuda Social Argentina Junio 2020.

[43] Droit-Volet S, Wearden JH. Experience Sampling Methodology reveals similarities in the experience of passage of time in young and elderly adults. Acta Psychologica. 2015 Mar 1;156:77–82. doi: 10.1016/j.actpsy.2015.01.006 25701720

[44] Pierce M, Hope H, Ford T, Hatch S, Hotopf M, John A, et al. Mental health before and during the COVID-19 pandemic: a longitudinal probability sample survey of the UK population. The Lancet Psychiatry. 2020 Oct 1;7(10):883–92. doi: 10.1016/S2215-0366(20)30308-4 32707037 PMC7373389

[45] Behm DG, Carter TB. Effect of Exercise-Related Factors on the Perception of Time. Frontiers in Physiology. 2020 Jul 6;11:770. doi: 10.3389/fphys.2020.00770 32733275 PMC7357302

[46] Levine RN. A geography of time: On tempo, culture, and the pace of life. Basic Books; 2008 Aug 1.

[47] Hall ET, Hall T. The silent language. Anchor books; 1959.

[48] Nonis SA, Teng JK, Ford CW. A cross-cultural investigation of time management practices and job outcomes. International Journal of Intercultural Relations. 2005 Jul 1;29(4):409–28.

[49] Persing DL. Managing in polychronic times. Journal of Managerial Psychology. 1999 Sep 1.

[50] Schwartz SH. Cultural value orientations: Nature and implications of national differences. Moscow: Publishing house of SU HSE. 2008 Apr 1.

[51] Friedman WJ, Janssen SM. Aging and the speed of time. Acta Psychologica. 2010 Jun 1;134(2):130–41. doi: 10.1016/j.actpsy.2010.01.004 . 20163781

[52] Janssen SM, Naka M, Friedman WJ. Why does life appear to speed up as people get older?. Time & Society. 2013 Jul;22(2):274–90.

[53] Wittmann M, Lehnhoff S. Age effects in perception of time. Psychological Reports. 2005 Dec;97(3):921–35. doi: 10.2466/pr0.97.3.921-935 . 16512313

[54] Winkler I, Fischer K, Kliesow K, Rudolph T, Thiel C, Sedlmeier P. Has it really been that long? Why time seems to speed up with age. Timing & Time Perception. 2017 May 6;5(2):168–89.

